# Can Industrial Structural Adjustment Improve the Total-Factor Carbon Emission Performance in China?

**DOI:** 10.3390/ijerph15102291

**Published:** 2018-10-18

**Authors:** Zhonghua Cheng, Xiai Shi

**Affiliations:** 1 China Institute of Manufacturing Development, Nanjing University of Information Science & Technology, Nanjing 210044, China; shixiai@seu.edu.cn; 2 School of Management Science and Engineering, Nanjing University of Information Science & Technology, Nanjing 210044, China; 3 Reading Academy, Nanjing University of Information Science & Technology, Nanjing 210044, China; 4 School of Economics and Management, Southeast University, Nanjing 210096, China

**Keywords:** structural adjustment, carbon emission performance, meta-frontier, dynamic spatial panel

## Abstract

How to improve the industrial total-factor carbon emission performance (TCPI), or total-factor carbon productivity, through industrial structural adjustment, is crucial to China’s energy conservation and emission reduction and sustainable growth. In this paper, we use a dynamic spatial panel model to empirically analyze the effect of industrial structural adjustment on TCPI of 30 provinces in China from 2000 to 2015. The results show that most of the provinces with high TCPI are located in the eastern coastal areas, while the provinces with relatively low TCPI are to be found in the central and western regions. The spatial auto-correlation tests show that there are significant global spatial auto-correlation and local spatial agglomeration characteristics in TCPI. The regression results of the dynamic spatial panel models show that at the national level, the structure of industrialization, the industrial structure of heavy industrialization, the coal-based energy consumption structure and the endowment structure have significant negative effects on the improvement of TCPI. The expansion of industrial enterprise scale, on the other hand, is conducive to an improvement in TCPI while the effects of foreign direct investment (FDI) structure and ownership structure on TCPI are not significant. At the regional level, there are certain differences in the effects of different types of industrial structural adjustment on TCPI.

## 1. Introduction

Since the reform and opening up, China’s economy has achieved rapid development, but it is accompanied by increasing environmental pollution. In 2012, China’s carbon emissions accounted for as much as 28.8% of the world’s total [1], and international calls for China to take mandatory carbon emission reduction measures have today become ever louder. As the largest carbon emitter in the world, China is actively participating in the framework of international climate cooperation and taking various measures to reduce carbon emissions. At the 2009 World Climate Conference, the Chinese government announced that the carbon emissions per unit of gross domestic product (GDP) would be reduced by 40–45% by 2020 relative to 2005 levels. The 13th Five-Year plan also explicitly put forward two binding targets that the energy consumption per unit of GDP, and the carbon emissions per unit of GDP would be reduced by 15% and 18% respectively by 2020 relative to 2015 levels.

However, China’s economic development level and social welfare level are relatively low, and the goal of pursing economic growth cannot be changed in a short time. Therefore, China has been adopting a relative carbon reduction strategy to reduce carbon emissions, that is, continuously improving carbon emission performance. China has been undergoing a rapid industrialization and is in middle stage of industrialization recently. At this stage, industrial development presents typical characteristics of high energy consumptions and high pollutant emissions. Although the secondary industry contributed 40.1% to China’s GDP between 2000 and 2015, its energy consumption and carbon emissions accounted for appropriately 67.9% and 84.2% of the national total, respectively [2]. How to improve industrial carbon emission performance is thus crucial to China’s economic growth, energy conservation, and emission reduction.

At present, both governments and academia have proposed improving carbon emission performance by adjusting economic structure and accelerating technical progress. Through structural reform, China is trying to promote both a greener industrial landscape and more sustainable development. We cannot help asking whether these structural adjustments can really improve industrial carbon emission performance. What structural adjustment plays a major role in improving industrial carbon emission performance? By looking at this question, we can better know how industrial structure can be adjusted to enhance green and sustainable development.

## 2. Literature Review

The theory of Chenery’s industrialization stages predicts that changes in industrial structure can have important effects on economic growth [3]. This is mainly because there are significant differences in productivity levels and growth rates between various industrial sectors—therefore, when an energy factor is transferred from sectors with low productivity or low productivity growth rates to sectors with high productivity or high productivity growth rates, this will promote an improvement in total industrial productivity. The balance of the total productivity growth rate above the weighted sum of each sector’s productivity growth rate is the contribution of industrial structural change to productivity growth [4]. With the increase in environmental problems, scholars have begun to introduce environmental factors into their ‘Structural Bonus’ research, and analyze the effects of structural changes on economic growth under environmental constraints.

Grossman and Krueger [5] (1995) put forward the theory of three effects of international trade on environment, namely, scale effect, structural effect and technical effect. It is widely adopted to study the effect of structural adjustment on environment. This theory holds that scale effect, structural effect and technical effect brought by economic growth together determine the effect of economic growth on environmental pollution. In the early stage of economic growth, scale effect is greater than structural effect and technical effect, and scale effect is dominant, thus aggravating environmental pollution. With the improvement of economic development level, structural effect and technical effect caused by economic growth are gradually greater than scale effect, and structural and technical effects are dominant, thus alleviating environmental pollution [6]. It shows that there is an inverted “U” relationship between economic growth and environmental pollution, verifying the environmental Kuznets curve (EKC) to some extent [7].

The vast majority of the literature uses carbon intensity to measure carbon emission performance and analyzes the effects of structural changes on that performance. The research methods used in the literature can be roughly divided into two categories: Factor decomposition methods and econometric analysis methods. Most early studies would decompose carbon intensity into several key factors using factor decomposition methods, including factors such as economic development, industrial structure and technological levels, and then analyze the relative effects of these factors on changes in carbon intensity. Because the factor decomposition method is both clear and concise, its calculation simple, and its decompositions easily observed, controlled and interpreted, it has been widely used by scholars. The Log-Mean Divisia Index (LMDI) [8,9,10], Structural Decomposition Analysis (SDA) [11,12,13] and optimization model [14,15] are often used to analyze the relationship between industrial structural adjustment and energy conservation, emission reduction and sustainable growth. There are, however, significant differences in the conclusions.

Most scholars believe that structural change is conducive to reducing carbon intensity. Fan et al. [16] (2007) used the LMDI method to decompose China’s carbon intensity from 1980 to 2003; their results showed that the main reason for the decrease in carbon intensity was the decrease in industrial energy consumption intensity. Zhang [17] (2009) applied the SDA method to analyze how industrial changes impacted carbon intensity in China; the results showed that changes in industrial structure, changes in aggregated sector structure within industries and within the manufacturing sector mix in the final demand, and changes in input mix can each decrease carbon intensity. Yi et al. [18] (2016) used the index decomposition method to analyze the driving factors in decreasing China’s carbon intensity from 2005 to 2020; the results showed that decreases in the proportion of secondary industry were conducive to reducing carbon intensity. Zhang et al. [9] (2016) used the LMDI method to analyze the driving factors of China’s carbon intensity from 1995 to 2012; the results showed that reduction in the proportion of industrial energy consumption was the main driving force for the reduction of carbon intensity.

Some scholars believe that the effect of structural change on carbon intensity is not significant. Liu et al. [8] (2015) used the LMDI method to decompose China’s industrial carbon intensity from 1996 to 2012; the results indicated that the reduction of energy intensity was the main contributor to the decrease of carbon intensity, while the effect of structural change on carbon intensity was not significant. Wang et al. [19] (2017) used the SDA method to analyze the driving factors that go into reducing global carbon intensity; the results showed that an improvement in energy efficiency was the main driving force for the decrease of carbon intensity, while the effect of structural change on carbon intensity was not significant.

Some scholars have even found that structural change has increased carbon intensity. Tan et al. [20] (2011) used the LMDI method to decompose China’s carbon intensity from 1998 to 2008; their results showed that structural change was not conducive to reducing carbon intensity. Gonzalez and Martinez [21] (2012) used the LMDI method to decompose Mexico’s carbon intensity from 1965 to 2010; their results showed that structural change increased carbon intensity to a certain extent.

Although the factor decomposition method is both relatively simple and clear and is able to calculate the contribution of related factors based on decomposition identity, it does have some deficiencies. Firstly, the factor decomposition method can only explain the pollutant flow change, and cannot explain the stock change, so this method is more applicable to explain situations where there is small pollutant stock and large pollutant flow change; the capacity to explain China’s situation with a lager pollutant stock and small pollutant flow change is relatively weak. Secondly, identity transformation may push many factors beyond consideration, so it is impossible to analyze the effects of these factors on pollutant reduction; this precludes an in-depth study into all the driving factors and mechanisms of pollutant reduction. Thirdly, not only are the economic and statistical meanings in factor decomposition methods relatively weak, but the decomposition process also often has a certain subjectivity, which may lead to fuzzy or irrational research conclusions. Fourthly, both how the decomposition method data is defined and how the data is partitioned greatly influence the results of carbon emission intensity factor decomposition [22].

Considering the shortcomings and deficiencies of the factor decomposition method, many scholars in later studies have turned to using econometric models to analyze the effect of structural changes on carbon intensity. Compared to factor decomposition methods, econometric analysis is relatively flexible and considers more influencing factors. It can also effectively use statistical and econometric methods to perform the theoretical tests, and thus has a strong theoretical basis in economics and statistics. Econometric analysis models include both cross section models and panel models. Because panel models can increase sample size, reduce collinearity between variables, control individual heterogeneity of samples, as well as improve reliability and validity of estimates, they have been popularly used in the area of carbon emissions [23,24].

In 2014, Zheng et al. [25] (2014) adopted a dynamic spatial panel to identify the determinants of provincial carbon intensity in China; the results showed that carbon intensity was positively associated with secondary-sector share. That same year, Yang et al. [26] (2014) used panel-corrected standard error estimates to analyze the effect of different factors on industrial carbon intensity in China; their results showed that industrial carbon intensity was positively associated with the six most energy intensive sectors in terms of total industrial output value. Zhao et al. [27] (2014) used spatial panel data models to analyze the drivers of carbon intensity in China; the results showed carbon intensity was positively affected by the structure of energy consumption. Hao et al. (2015) used the generalized moment method (GMM) estimators to test the convergence of carbon intensity in China; the results showed that the value added to GDP by secondary industry was not conducive to the convergence of carbon intensity. Wang et al. [28] (2016) used a regression analysis to study the key factors influencing carbon intensity in China; their results showed that the proportion of secondary industry was beneficial to a reduction in carbon intensity. Long et al. [29] (2016) used spatial panel data models to examine the factors influencing industrial carbon productivity; their results showed that industrial scale structure exerted a positive effect on industrial carbon productivity, but industrial energy consumption structure had a significantly negative effect on industrial carbon productivity. Cheng et al. [30] (2018) used dynamic spatial panel models to analyze the effects of industrial structure on carbon intensity in China; their results showed that the upgrading and optimization of industrial structure were conducive to reducing carbon intensity.

The above studies have provided us with a number of results, but there are still some areas for further study. The above literature mainly analyzes the carbon reduction effect from a single-factor perspective, but the main problem with single-factor carbon emission performance is that it only considers output; it ignores the effects of input factors such as capital, labor, energy etc., and their mutual substitution on carbon emission performance [31,32]. In fact, carbon emission performance is the result of the combination of capital, labor and energy, and shows a clear total-factor characteristic. It is thus more appropriate to adopt the total-factor carbon emission performance index. There are, however, significant differences in the definition of structural change in the various studies. Most studies have performed their analyses from either the perspective of the three major structures of industry or on energy consumption. Because previous studies have focused more on all industries, so the pertinence of the conclusions in these studies is not strong. A few studies have focused on secondary industry [29], but the elaboration of industrial structure is limited to light and heavy industrial structure. Factors as industrial scale structure, endowment structure, ownership structure etc., are often ignored.

In view of the foregoing, this paper makes the following contributions: First, we measure the total-factor carbon emission performance using non-radial directional distance function (NDDF) and meta-frontier method. Compared to previous measurement methods, this method fully considers the technology heterogeneity, making the results more accurate and reliable. Second, we focus on secondary industry, and subdivide industrial structure into light and heavy structure, scale structure, endowment structure, ownership structure, energy consumption structure and FDI structure. Compared to previous studies, the structural variables we consider are more comprehensive, and the results also show that different structural variables have different effects on TCPI. Third, we use a dynamic spatial panel model that incorporates the spatial and dynamic effects of TCPI to empirically analyze the effects of these different types of structural changes on TCPI and their regional differences. The results show that TCPI presents obvious spatial and dynamic effects, and ignoring these effects may lead to errors in estimation and analysis.

## 3. Materials and Methods

### 3.1. Dynamic Spatial Panel Model

In this part we establish a dynamic spatial panel model to analyze the effects of structural changes on TCPI. Previous studies have shown that industrial structure and technological level are important factors affecting TCPI, because the TCPI is essentially a total-factor productivity (TFP) [4,29]. In China, a country with strong government intervention, environmental policy factors will inevitably have an important impact on TCPI [30,33]. We thus construct the following basic econometric model of the factors affecting TCPI as Equation (1):

ln*TCPI_it_* = *α* + *δ*ln*Str_it_* + *φ*ln*Tech_it_* + *ϕ*ln*Env_it_* + ε_it_ (1)

In Equation (1), *Str* denotes industrial structure, *Tech* denotes technological level, *Env* denotes environmental policy and ε denotes random error term. Model (1) is an ordinary static panel model, which does not consider the spatial relationship between regional economic development or the dependence of regional economic development on previous accumulation. In fact, with the rapid development of transport infrastructure and network communication technology, there is a strong spatial relationship between provinces in economic development terms, and CO_2_ can flow freely across the region, indicating that there is a spatial effect between regions in the TCPI. At the same time, there is significant path dependence in regional economic development. The previous accumulation is necessarily manifested by technological level, industrial structure, human capital, infrastructure etc., which can affect economic activities in both the current period and subsequent periods, indicating that there is a spatial effect in the TCPI. We, thus, incorporate the spatial and dynamic effects of the TCPI to construct the following dynamic spatial panel model as Equation (2).

(2) $$\ln TCPI_{it}=\tau \ln TCPI_{i\left(t-1\right)}+\rho \sum_{j=1}^{N}W_{ij}\ln TCPI_{jt}+\alpha +\delta \ln Str_{it}\;+\phi \ln Tech_{it}+\varphi \ln Env_{it}+\eta_{i}+\nu_{t}+\varepsilon_{it}\;\varepsilon_{it}=\lambda \sum_{j=1}^{N}W_{ij}\varepsilon_{jt}+\mu_{it}$$

In Equation (2), τ denotes the regression coefficient of first-order lag of TCPI and reflects the dynamic effect. ρ and λ respectively denote the regression coefficients of spatial lag and spatial error, and reflect the spatial effects. *W_ij_* denotes the geographic distance spatial weight matrix. $\eta_{i}$, $\upsilon_{t}$ and $\varepsilon_{it}$ denote different dimensions of random interference influencing TCPI, respectively.

### 3.2. Variable Description and Data Sources

Depending on the availability and validity of data, we selected statistical data from 30 provinces in mainland China from 2000 to 2015, because during these periods, China has experienced three Five-Year plan periods: The 10th Five-Year, the 11th Five-Year, and the 12th Five-Year. Tibet was not included in the analysis due to lack of data. The data are derived from China Industry Economy Statistical Yearbook (2001–2016), China Energy Statistical Yearbook (2001–2016), China Statistical Yearbook (2001–2016).

#### 3.2.1. Explained Variable: Industrial Total-Factor Carbon Emission Performance (TCPI)

We use the non-radial directional distance function (NDDF) and meta-frontier methods proposed by Oh [34] (2010) and Oh and Lee [35] (2010) to measure the TCPI, and then decompose the TCPI into three components: Technological efficiency change index (EC), best practice gap change index (BPC) and technological gap change index (TGC). More detailed measurement and decomposition process can be referred to [2,36,37,38,39].

The key to the measure of TCPI is the selection of input and output variables, and the following is a specific description of each of the input and output variables. (1) Input variables: With respect to the input index, we use three variables—capital, labor and energy. Capital is represented by the amount of capital stock, which can be calculated by the perpetual inventory method [40]. We use the average number of industrial workers and energy consumption (converted to 10,000 tons of standard coal) to measure labor and energy, respectively. (2) Desirable output variable: Desirable output is derived from the main business income of industrial enterprises above designated size deflated by GDP price indices in 2000. (3) Undesirable output variable (C): We calculate carbon emissions using the calculation method provided by IPCC and the specific calculation process can be seen from [30]. The data of capital, labor and desirable output are derived from China Industry Economy Statistical Yearbook and China Statistical Yearbook, and the data of energy and undesirable output are derived from China Energy Statistical Yearbook.

#### 3.2.2. Core Explanatory Variables: Industrial Structure (*Str*)

In order to further analyze the effects of industrial structure changes on TCPI, we subdivide industrial structure as follows:

(1) The industry and service structure (IS). As we all know, the second industry is the major industry in China’s energy consumption and carbon emissions, and the rapid development of the second industry can also inhibit the development of the third industry. Thus, an increase in the IS is not conducive to an improvement in TCPI. In the paper, we use the proportion of main business income of Second industry to main business income of third industry to measure IS. These data are derived from China Statistical Yearbook.

(2) Industrial light and heavy structure (LH). In general, according to the characteristics of heavy industry, heavy industrialization is often accompanied by high energy consumption and high pollutant emissions, which is not conducive to an improvement in TCPI. In the paper, we use the proportion of main business income of heavy industrial enterprises to main business income of industrial enterprises above a designated size to measure LH. These data are derived from China Industry Economy Statistical Yearbook.

(3) Industrial scale structure (SC). Generally speaking, large and medium-sized industrial enterprises have strong technological innovation and management capabilities, and more easily obtain increasing returns to scale; this is conducive to an improvement in TCPI. We use the proportion of main business income of large and medium-sized industrial enterprises to main business income of industrial enterprises above a designated size to measure SC. These data are derived from China Industry Economy Statistical Yearbook.

(4) Industrial ownership structure (OW). At the micro level, there are significant differences in production efficiency, resource allocation and utilization, management mechanisms etc. between industrial enterprises with different ownership structures. This will inevitably have an important impact on TCPI. We use the proportion of main business income of state-owned and state-controlled industrial enterprises to main business income of industrial enterprises above a designated size to measure OW. These data are derived from China Industry Economy Statistical Yearbook.

(5) Industrial endowment structure (EN). When looking at the totality of factors, because there is a certain mutual substitution or complementary relationship between capital, labor and energy, endowment structure can affect carbon emissions. It does this by influencing the changes in total energy consumption, thereby affecting the TCPI. We use the ratio of industrial capital to labor to measure EN. If the value is low, it indicates that the regional industrial type tends to be labor-intensive; otherwise it tends to be capital-intensive. Since the increase in the ratio of capital to labor means capital deepening, we can use this variable to further analyze the effect of capital deepening on TCPI. These data are derived from China Industry Economy Statistical Yearbook.

(6) Industrial energy consumption structure (ECS). Because there are big differences in thermal efficiencies between coal and oil and natural gas, and because the amount of carbon and pollution resulting from coal consumption is higher than for the other energies, a coal-dominated energy consumption structure is not conducive to an improvement in TCPI. We use the percentage of industrial coal consumption (converted to standard coal) to total industrial energy consumption to measure ECS. These data are derived from China Energy Statistical Yearbook.

(7) Industrial FDI structure (FDI). Foreign direct investment on industry has two effects. On the one hand, FDI can bring advanced technology and management, thereby improving the technological and quality levels of domestic enterprises through spillovers of knowledge and technology. On the other hand, strict environmental regulation policies in foreign countries may encourage the transfer of enterprises with high energy consumption and high pollutant emissions to China, which is not conducive to China’s low-carbon development. We use the proportion of main business income of foreign invested industrial enterprises to main business income of industrial enterprises above a designated size to measure FDI. These data are derived from China Industry Economy Statistical Yearbook.

#### 3.2.3. Control Variables

(1) Technological level (*Tech*). Scientific and technological innovation and technical progress are the fundamental driving forces for industrial green development. They can help industrial enterprises improve factor utilization and promote the intensive, rational and effective utilization of resources. Because technological level is most closely related to research and development (R&D), we use the internal R&D expenditure of industrial enterprises above a designated size to measure Tech. These data are derived from China Statistical Yearbook.

(2) Environmental policy (*Env*). Environmental regulation policy can exert an important influence on TCPI through the innovation compensation and compliance cost effects. We use the proportion of completed investment of industrial pollution control projects to main business income of industrial enterprises above a designated size to measure *Env*. These data are derived from China Statistical Yearbook and China Energy Statistical Yearbook. Table 1 lists the descriptive statistics of input and output variables for the whole of China and its three regions, and Table 2 lists the descriptive statistics of the panel data.

## 4. Results

### 4.1. The Meta-Frontier Total-Factor Carbon Emission Performance Since the 21st Century

As can be seen from Table 3, the TCPI of all the provinces in the study period are greater than 1, showing that the carbon emission efficiency of all the provinces has increased, albeit, to differing extents. This indicates that China’s policies on energy saving and emission reduction have been beneficial. Chongqing has the highest TCPI, and its average value is as high as 1.2257. In terms of its decomposition, we find that the improvement in technological efficiency and technical progress and the decrease in technology gap have jointly promoted an improvement in TCPI; technical progress has played a major promoting role. Qinghai has the lowest TCPI, and its average value is only 1.0225. In terms of its decomposition, although its technical progress is conducive to an improvement in TCPI, deterioration in technological efficiency and expansion of technology gap have together inhibited an improvement of TCPI to a certain extent.

In the study period, improvement in technological efficiency is conducive to an improvement of TCPI in 21 provinces, with the improvement in Hubei the strongest. The deterioration in technological efficiency has however inhibited an improvement in TCPI in 8 provinces, with the inhibiting effect in Xinjiang the strongest. Technical progress is conducive to the improvement of TCPI in all the provinces, with the positive effect in Inner Mongolia the strongest, and Hunan the weakest. The decrease of technology gap is conducive to the improvement of TCPI in 9 provinces, with the effect on Guizhou the strongest. The expansion of the technology gap has inhibited improvements in TCPI in 15 provinces, with the inhibiting effect on Hubei the strongest.

From the point view of each region, the TCPI in the eastern region is the highest, followed by the central region, with the lowest in the western region. In terms of its decomposition, the improvements in TCPI in the eastern and central regions are mainly driven by improvements in technological efficiency and technical progress. Technical progress has played a major promoting role, although the expansion of the technological gap has inhibited the improvement in TCPI to a certain extent. The improvement of TCPI in the western region is mainly driven by technical progress, while the deterioration in technological efficiency has somewhat inhibited the improvement in TCPI.

### 4.2. Spatial Auto-Correlation Tests

The spatial auto-correlation is inconsistent with the dependence and randomness of the basic assumptions in traditional statistics, making traditional statistical methods no longer valid [41]. Therefore, it is necessary to test whether there is the spatial auto-correlation in statistical data. We first use the *Moran’s I* index to test the global spatial auto-correlation of TCPI as Equation (3).

(3) $$Moran’s\;I=\frac{\sum_{i=1}^{N}\sum_{j=1}^{N}W_{ij}\left(y_{i}-\bar{y}\right)\left(y_{j}-\bar{y}\right)}{S^{2}\sum_{i=1}^{N}\sum_{j=1}^{N}W_{ij}}$$

In Equation (3), *y* denotes the TCPI in province *i*, $\bar{y}$ and $S^{2}$ denote its mean and variance respectively. We also use the standard statistic *Z* to test the significance of the *Moran’s I* and Table 4 lists the results of *Moran’s I* index and its significance level from 2000 to 2015.

**Figure 1:**
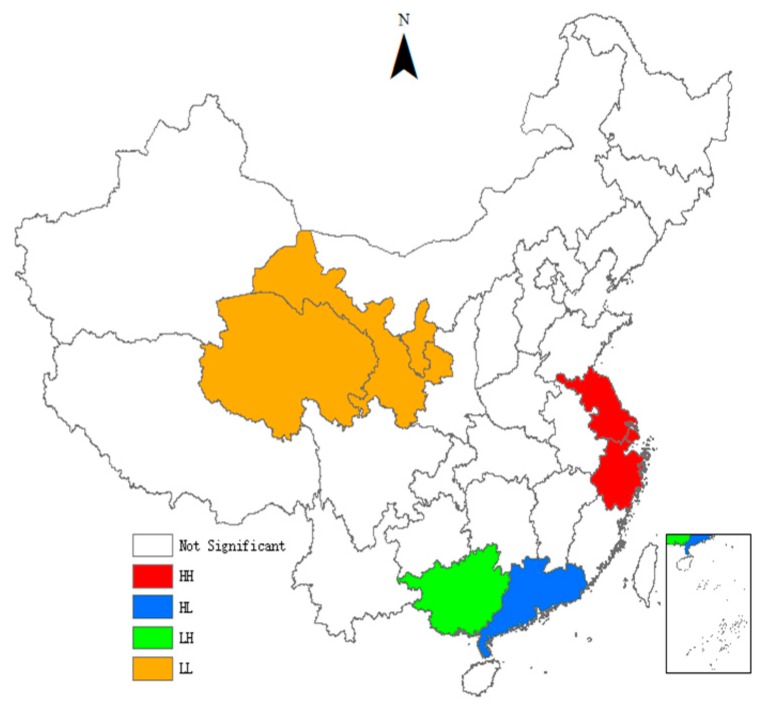
LISA agglomeration between 2000 and 2001.

**Figure 2:**
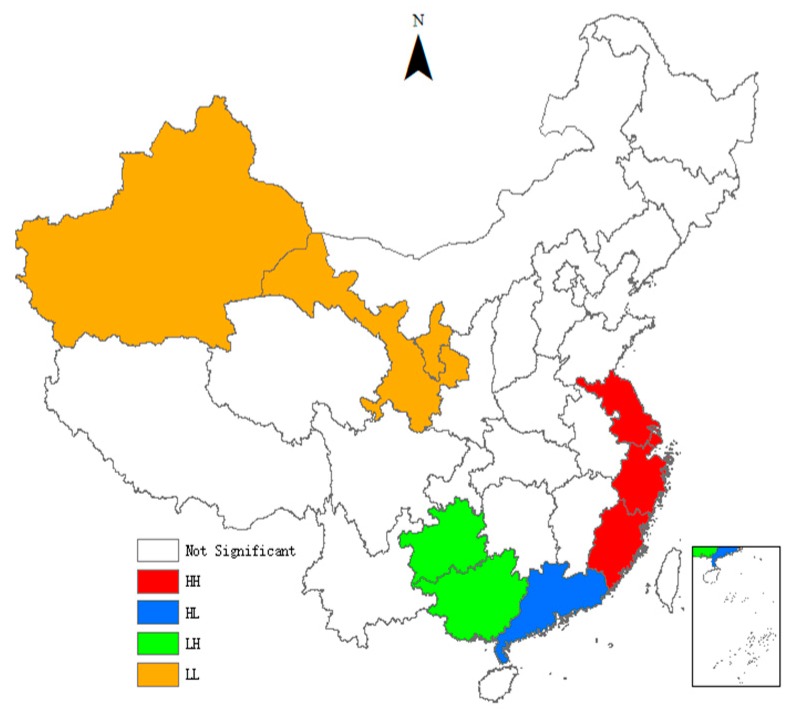
LISA agglomeration between 2005 and 2006.

**Figure 3:**
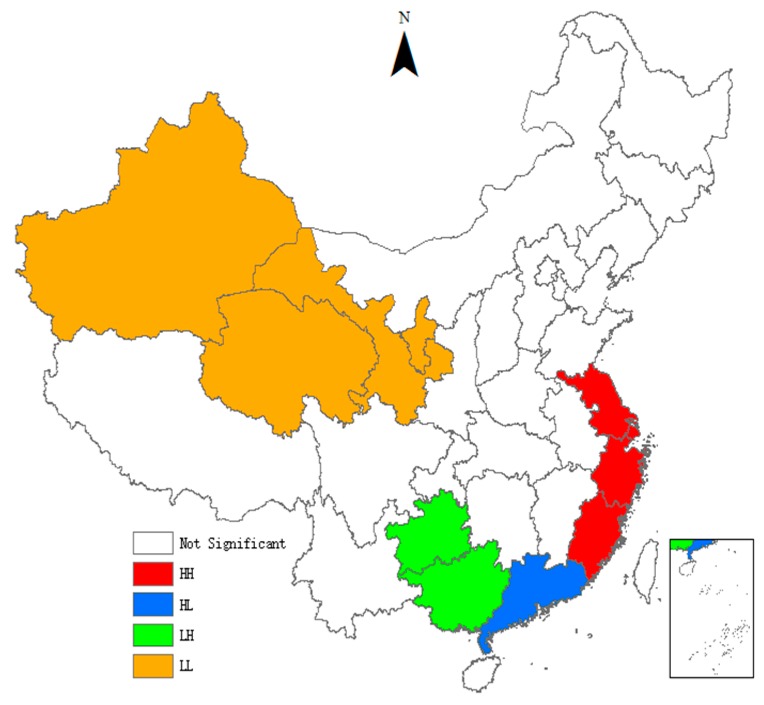
LISA agglomeration between 2010 and 2011.

**Figure 4:**
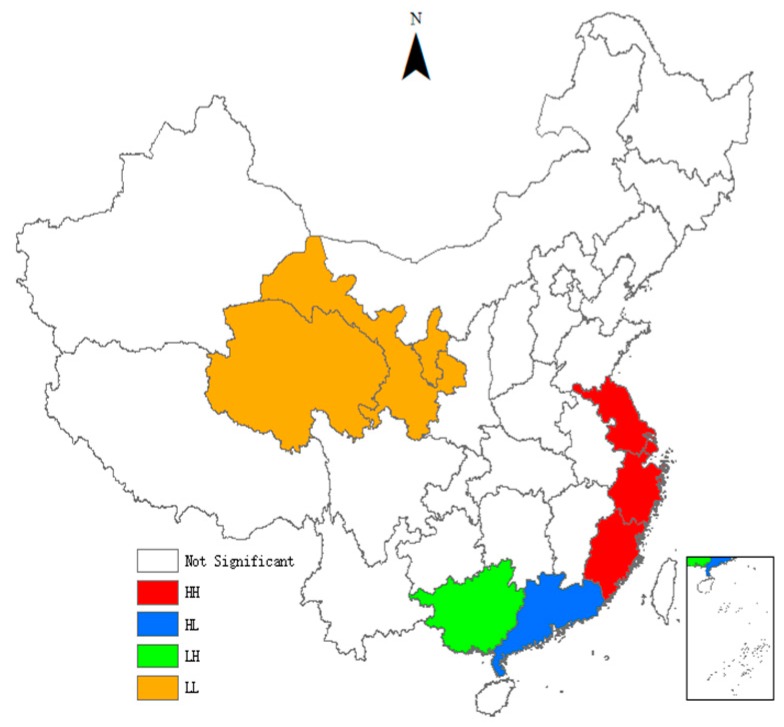
LISA agglomeration between 2014 and 2015.

As can be seen from Table 4, the *Moran’s I* indices of the TCPI are all significantly positive, indicating that there is a significant global spatial positive auto-correlation in the TCPI between different provinces. It means that provinces with similar TCPI have significant spatial agglomeration effect. With the passage of time, the *Moran’s I* indices show an inverted ‘U’ trend, indicating that the spatial agglomeration effect first enhances and then gradually weakens. We also use the local indicators of spatial association (LISA) to test the spatial heterogeneity. To visualize local spatial autocorrelation, we draw the LISA agglomeration maps using ArcGIS. Figure 1, Figure 2, Figure 3 and Figure 4 present LISA agglomeration maps of TCPI for 2000–2001, 2005–2006, 2010–2011 and 2014–2015 respectively.

As can be seen from the four LISA agglomeration maps, the High-High agglomeration areas of provincial TCPI from 2000 to 2015 were mainly concentrated in Shanghai, Zhejiang, Jiangsu and Fujian provinces. This indicates that the TCPIs in these areas were high, the spatial correlation between these provinces was strong, and these areas had formed a local spatial high-value group. Guangdong was located in the High-Low agglomeration areas in these four years, indicating that although the TCPI of Guangdong was high, its driving effect on the surrounding provinces was not enough. There are two main reasons for this phenomenon. On the one hand, Guangdong has a high economic development level and has a strong siphon effect on the innovative resources and talents in surrounding provinces. On the other hand, the spatial spillover effects of knowledge and technology brought by the agglomeration of innovative resources have a locality, and it gradually weakens with the increase of geographical distance. Furthermore, the serious market segmentation between provinces also inhibits the spatial spillover of knowledge and technology. Guangxi and Guizhou intermittently appeared in the Low-High agglomeration areas, indicating that the TCPI of these two provinces was low, but the TCPI of the surrounding provinces was high. This means that Guangxi and Guizhou are not driven by the high TCPI provinces that surround them, and they belong to the hollow zone. This also means that the interconnections between Guangxi, Guizhou and the surrounding provinces are weak, and there is a lack of ‘pass, help and band’ effect between them. The Low-Low agglomeration areas were mainly concentrated in Ningxia, Gansu, Qinghai and Xinjiang provinces from 2000 to 2015. This indicates the TCPIs in these areas are low, the spatial correlation between these provinces is weak, and the radiation from provinces with high levels is also very limited.

## 5. Discussion

### 5.1. Analysis of Regression Results at the National Level

In order to demonstrate and verify the superiority and effectiveness of the dynamic spatial panel model, we simultaneously adopt the ordinary static panel model, the ordinary dynamic panel model and the static spatial panel model for comparison. We use the feasible generalized least squares (FGLS) method and the generalized method of moments (GMM) method to estimate the ordinary static panel model and the ordinary dynamic panel model respectively. For the estimation of static spatial panel model, we use the Maximum Likelihood (ML) method proposed by Elhorst [42] (2005). For the estimation of dynamic spatial panel model, we use the spatial system GMM method proposed by Kukenova and Monteiro [43] (2009) and Jacobs et al. [44] (2009). The whole process is calculated by Stata 13.0 and the results are shown in Table 5.

As can be seen from Table 5, the coefficient of spatial spillover effect in the results of dynamic spatial panel model is significantly positive, indicating that provincial TCPI has a significant spatial spillover effect. But neither the ordinary static panel model nor the ordinary dynamic panel model considers the spatial spillover effect of TCPI, thereby may leading to errors in estimation. Meanwhile, the coefficient of dynamic effect in the results of dynamic spatial panel model is significantly positive, indicating that provincial TCPI has a significant dynamic effect. But neither the ordinary static panel model nor the static spatial panel model considers the dynamic effect of TCPI, thereby may leading to errors in estimation. We can also find that the regression results of the dynamic spatial panel model are superior to those of the former, and thus select dynamic spatial panel model as our final interpretation model.

From the regression results of the dynamic spatial panel model, we note that the coefficient of IS is negative at the 1% significance level, indicating that the structure of industrialization has significantly inhibited the improvement of TCPI. This is mainly because that when the proportion of the second industry in a region is higher, its third industry, especially the producer service industry develops slowly. Then its producer service industry cannot better promote industrial transformation and upgrading and green development through scale effect, specialization effect, knowledge and technology spillover effect, and competition effect.

The coefficient of LH is significantly negative, indicating that industrial structure of heavy industrialization has significantly inhibited the improvement of TCPI. This is mainly because, compared to light industry, heavy industry belongs to the high energy consumption and high pollutant emission industry category. Considering the coal-dominated industrial energy consumption structure, energy efficiency and carbon emission efficiency in heavy industry is lower than that of light industry and this has had a significant negative effect on the improvement of TCPI.

The coefficient of SC is positive at the 5% significance level, indicating that expansion of industrial enterprise scale is conducive to an improvement of TCPI. We deduce two main reasons for this conclusion. On the one hand, the expansion of enterprise scale can increase the profit base of enterprises, help enterprises transform, upgrade and update their equipment and technologies and also help enterprises introduce new technologies, thereby improving their technological level. On the other hand, the expansion of enterprise scale is more conducive for enterprises to achieve increasing returns to scale and marginal cost reduction, thus improving their energy efficiency and carbon emission efficiency.

The coefficient of OW is not significant, indicating that the effect of enterprise ownership structure on TCPI is not significant. With the deepening of the reform process of state-owned enterprises, the vitality and market competitiveness of some state-owned enterprises has been released. Their TCPIs have improved, relying on the advantages of economies of scale and innovative research and development. However, the institutional mechanism of state-owned enterprises in China is still not very sound, the economic growth mode is still relatively extensive, and redundant construction and overcapacity are still very serious. These are not conducive to improvements in TCPI. The interaction between these two effects determines the significance of industrial ownership structure, which is not initially obvious.

The coefficient of EN is significantly negative, indicating that capital deepening is not conducive to the improvement of TCPI. As we all know, the increase of the ratio of capital to labor indicates that economic structure has transformed from labor-intensive to capital-intensive. Generally speaking, the labor-intensive industry tends to be in the light pollution industries and the capital-intensive industry tends to be in the heavy pollution industries. China is now in the middle stage of industrialization, and a sustained rise in the ratio of capital to labor is mainly dependent on the extensive expansion of the scale of industrial enterprise. Capital deepening has made capital continuously flow to such heavy industries as steel, cement and chemicals, which not only deteriorates environmental quality, but also reduces the TCPI.

The coefficient of ECS is significantly positive, indicating that the coal-dominated energy consumption structure has significantly inhibited TCPI improvement. This is mainly because, compared to oil and natural gas, the energy consumption and pollutant emissions per unit of output of coal are high, but its thermal efficiency is low. Therefore, changing the coal-dominated energy consumption structure is an important way for China to improve TCPI. 

The effect of FDI structure on TCPI is not significant, because FDI in China’s industry is mainly concentrated in labor-intensive and resource-intensive industries. Although FDI can bring a spillover of knowledge and technology to a certain extent, it causes consumption of large amounts of energy with consequent large carbon emissions, thus making the effect of FDI on the improvement of TCPI not significant.

Comparing the coefficients and significances of these structural variables, we can find that among the carbon emission performance of structural adjustments, the effect of industrial light and heavy structure is the strongest, followed by industrial energy consumption structure, the third is industrial scale structure and the last is industrial endowment structure. This means that in order to improve the industrial carbon emission performance, we should pay more attention to industrial light and heavy structure and industrial energy consumption structure.

As for the control variables, a more advanced technology level is conducive to an improvement in TCPI. This is mainly because technical progress can not only increase economic output, but can also reduce energy consumption, which is the essential source of any improvement in energy efficiency. Environmental regulation is also conducive to improvements in TCPI, indicating that the innovation compensation effect of environmental regulation is dominant in industry in China. In order to decrease the compliance costs, the regulated enterprises must transform, upgrade and update their equipment and technologies.

### 5.2. Analysis of Regression Results at the Regional Level

As China’s TCPI and industrial structure show significant differences in different regions, we have analyzed the effects of industrial structure changes on TCPI in the eastern, the central and the western regions. We still use a dynamic spatial panel model to estimate the data in different regions, and the results are shown in Table 6.

As can be seen from Table 6, the first-order lag coefficients of TCPI in the three regions are all positive at the 1% significance level, indicating that there are continuous dynamic effects of TCPI in different regions. Comparing their coefficients, we find that the eastern region shows the largest, followed by the central region and the western region, the smallest. This is mainly because the eastern region has high economic development levels, technical levels and carbon emission performance, so the improvement of TCPI is more dependent on previous foreshadowing and accumulation. Furthermore, the spatial lag coefficients of TCPI in the three regions are also all positive at the 1% significance level, indicating that there are significant spatial spillover effects of TCPI in different regions. Comparing their coefficients we find that the eastern region exhibits the largest, followed by the central region and the western region, the smallest. This is mainly because the eastern region has a better transportation infrastructure, has higher informatization levels, and has closer and more frequent economic and trade activities with neighboring provinces. This leads the eastern region to having a greater spatial spillover effect of TCPI between different provinces.

The regression results show that both the structure of industrialization, the industrial structure of heavy industrialization and the coal-dominated energy consumption structure have inhibited the improvement of TCPI in the eastern region. The expansion of enterprise scale is conducive to an improvement in TCPI, but the effects of enterprise ownership structure and FDI structure on improvement are not significant. Unlike the estimated results at the national level, capital deepening in the eastern region is conducive to an improvement in TCPI. This is probably because, although capital deepening makes industrial structure shift from labor-intensive to capital-intensive, the capital-intensive enterprises in the eastern region have higher technology levels, which not only offset the negative effect of capital deepening on resources and environment, but also bring improvements in energy efficiency.

For the central region, the regression results show that the structure of industrialization, the industrial structure of heavy industrialization, the coal-dominated energy consumption structure and capital deepening have all inhibited improvements in TCPI. The expansion of enterprise scale is conducive to improvements in TCPI, but the effect of FDI structure on TCPI improvements is not significant. Unlike figures for the national level, enterprise ownership structure for the central region is conducive to the improvement in TCPI, indicating that the reform of state-owned enterprises in the central region has achieved remarkable results. This can be mainly attributed to the central region has a high proportion of state-owned enterprises, and the reforms of state-owned enterprises and state-owned capital are carried out systematically and in depth. With the advantages of economies of scale and innovative research and development, the continuous reformation of state-owned enterprises has improved TCPI in the central region.

For the western region, the regression results show that the structure of industrialization, the industrial structure of heavy industrialization, the coal-dominated energy consumption structure and capital deepening have inhibited the improvement of TCPI and the effect of enterprise ownership structure on the improvement of TCPI is not significant. Unlike the results obtained at the national level, the effect of enterprise scale structure on improvements in TCPI in the western region is not significant. This is likely because the western region has a low proportion of large and medium-sized enterprises, which cannot give full play to the advantages of large enterprise scales in energy saving and emission reduction. The FDI structure has significantly inhibited the improvement of TCPI in the western region, probably because the western region has relatively relaxed environmental regulations, which has made it gradually become the pollution haven of FDI.

### 5.3. Robustness Test

In order to further test the robustness of the results, we use an economic distance spatial weight matrix to replace the geographic distance spatial weight matrix for retesting. We also use the dynamic spatial panel model to perform the regression. The economic distance spatial weight matrix can be constructed as Equation (4).

(4) $$W_{ij}^{e}=W_{ij}{}^{d}\cdot diag\left({\bar{Y}}_{1}/\bar{Y},{\bar{Y}}_{2}/\bar{Y},\cdots ,{\bar{Y}}_{N}/\bar{Y}\right)$$

In Equation (4), $W_{ij}^{e}$ and $W_{ij}{}^{d}$ denote the economic distance and geographic distance spatial weight matrices respectively, ${\bar{Y}}_{i}$ denotes the mean of actual GDP for province *i* in the study period, and $\bar{Y}$ denotes the mean of actual GDP for all the sample provinces in the study period. As can be seen from Table 7, although the coefficients of some control variables and spatial spillover effect, as well as their significance have increased or decreased to some extent, the estimated results of the core explanatory variables are consistent with the above conclusions, indicating that the results are reliable and robust.

## 6. Conclusions

In the paper we accurately measure the TCPI of 30 provinces in China, from 2000 to 2015, under a meta-frontier and total-factor analysis framework and then adopt a dynamic spatial panel model to empirically analyze the effect of industrial structural adjustment on TCPI. The results show that most of the provinces with high TCPI are located in the eastern coastal areas, while the central and western provinces have relatively low TCPI. There are also significant global spatial auto-correlation and local spatial agglomeration characteristics in TCPI. At the national level, the structure of industrialization, the industrial structure of heavy industrialization, the coal-based energy consumption structure and the endowment structure have significantly inhibited improvements in TCPI. The expansion of enterprise scales is conducive to the improvement of TCPI, but the effects of FDI and ownership structures on TCPI are not significant. At the regional level, there are certain differences in the effects of different types of industrial structural adjustment on TCPI. Based on the above conclusions, the following points are made.

(1) China should promote the development of the third industry, especially the producer service industry through a series of industrial polices, promote the large-scale, market-oriented and organized operation of producer service market, constantly reduce production costs and service risks and improve the level of specialized services. Furthermore, China should guide and drive manufacturing enterprises to gradually outsource some non-core productive services, focus on the core manufacturing links, such as technology research and product competitiveness enhancement, and effectively improve the technology level of manufacturing enterprises.

(2) China should accelerate the development of new manufacturing, actively implement the upgrading of heavy industries, and focus on the transformation, upgrading and updating of traditional industries, such as the automobile, steel, cement and chemical industries. For heavy industries, China should also gradually implement green manufacturing projects, promote the green management of product life cycles, and accelerate the construction of green manufacturing and green supply chain industry systems.

(3) China should speed up the reform of state-owned enterprises both internally and in the shareholding system, gradually improving the state-owned assets management system, modern enterprise management systems and corporate governance structures. For state-owned enterprises, China should also strengthen cost controls, product quality, operational efficiency and guarantee capability assessment, with the goal of making state-owned enterprises stronger, better and bigger.

(4) In the process of reforming state-owned enterprises, China should continue to strive for the reorganization and integration of more resources, using various acquisition methods, such as using its own funds, low cost debt, industry funds and mergers and acquisitions funds. These funds can be used to increase market mergers and acquisitions and gradually form industrial organizations with, at their core, large highly concentrated enterprise groups that exhibit a detailed division of labor and efficient cooperation.

(5) China should further deepen capital levels and encourage capital to invest in such areas as high-tech industries, energy conservation and environmental protection industries and strategic emerging industries. Furthermore, not only should China gradually establish promote a system with constraints on process, technology, energy consumption, environmental protection, quality and safety, but it should also strengthen industrial norms and access management, eliminate backward production capacity, and actively and steadily resolve overcapacity.

(6) China should optimize the development and utilization of coal, and vigorously promote clean and efficient use of coal. It is also important to vigorously develop renewable and new energy through scientific and technological innovation, and promote the optimization and upgrading of energy structure. In addition, China should construct modern energy storage, transportation network and intelligent energy systems, as well as implement cleaner production transformation in key industries, and speed up the implementation of alternative clean energy projects.

(7) China should further strengthen the screening and management of any foreign technology introduced into the country and restrict foreign enterprises investing in both high energy consumption and high pollution industries. China should encourage foreign enterprises to invest in R&D centers, high-tech industries, advanced manufacturing and energy conservation and environmental protection industries. It should also encourage foreign enterprises to perform the technological transformation and upgrading of traditional industries and encourage foreign enterprises to introduce core technologies and expand the technology spillover effect.

(8) China should strengthen R&D in industrial low-carbon technology, encourage enterprises to improve production technologies, upgrade processing equipment, become more energy efficient and aim for international standards of environmental protection. It should gradually realize the breakthrough into key areas for middle and high end products. Furthermore, China should strengthen manufacturing in areas, such as industrial energy conservation and environmental protection engineering technology and equipment, accelerate research and development and demonstrate and promote advanced energy conservation and environmental protection technology and equipment.

(9) China should gradually improve existing environmental regulations, raise both industrial pollutant emission standards and cleaner production evaluation indicators, and encourage each region to set more stringent pollution discharge standards depending on the situation. China should also establish an efficient environmental supervision and management system, strengthen inspections, supervision and management, and vigorously investigate environmental violations according to the law.

There are also some limitations in this paper, and these are mainly reflected in the following: Due to the availability and validity of data, we did not subdivide the second industry, so we cannot dig deep into the industrial heterogeneity of the effects of structural changes on TCPI. We will try to solve these problems in future research.

## Figures and Tables

**Table 1:** Descriptive statistics of input and output variables in China’s industrial sector.

Variable	Region	Mean	Std. Dev.	Min	Max
Capital (Unit: 100 million Yuan)	Whole China	15,519.26	17,826.92	394.84	107,061.70
	Eastern China	25,544.24	23,773.77	394.84	107,061.70
	Central China	12,402.87	10,284.41	1835.97	55,710.97
	Western China	7760.75	7693.80	511.12	40,401.38
Labor (Unit: Ten thousand)	Whole China	263.24	288.06	9.62	1568.00
	Eastern China	452.44	384.17	9.62	1568.00
	Central China	226.40	118.64	95.72	717.31
	Western China	100.83	76.13	13.48	397.81
Energy (Unit: Ten thousand tons of standard coal)	Whole China	3085.38	2444.94	119.94	13,237.40
	Eastern China	3797.53	3331.51	119.94	13,237.40
	Central China	3416.13	1689.31	782.44	7875.37
	Western China	2132.68	1299.62	201.43	5946.91
Desirable output (Unit: 100 million Yuan)	Whole China	17,368.34	24,773.01	174.75	147,074.50
	Eastern China	30,892.18	34,044.71	174.75	147,074.50
	Central China	14,075.54	14,367.16	896.87	73,365.96
	Western China	6239.27	7283.45	195.74	38,645.91
Undesirable output (Unit: Ten thousand tons)	Whole China	8382.44	6970.77	242.36	38,938.67
	Eastern China	10,412.24	9593.46	242.36	38,938.67
	Central China	9424.87	4791.35	2197.16	21,735.77
	Western China	5594.50	3398.83	493.55	14,494.58

**Table 2:** Descriptive statistics of panel data.

Variable	Obs	Mean	Std. Dev.	Min	Max
TCPI	450	1.139	0.300	0.503	2.191
IS	450	1.204	1.739	0.137	3.584
LH	450	74.479	10.701	42.376	95.684
SC	450	68.898	9.684	35.526	88.029
OW	450	45.501	20.696	10.069	90.142
EN	450	72.391	51.715	16.779	349.599
ECS	450	80.271	15.939	20.812	97.617
FDI	450	19.550	16.900	1.122	65.640
Tech	450	156.367	238.300	0.630	1520.550
Env	450	16.089	12.906	0.695	104.815

**Table 3:** Carbon emission performance and its decomposition in 2000–2015.

Group	Provinces	TCPI	EC	BPC	TGC
East	Beijing	1.2064	1.0555	1.1552	1.0000
East	Tianjin	1.1827	1.0545	1.1513	1.0000
East	Hebei	1.1685	1.0491	1.1233	0.9946
East	Liaoning	1.1241	1.0158	1.1215	0.9864
East	Shanghai	1.1366	0.9995	1.1373	1.0000
East	Jiangsu	1.1632	1.0125	1.1406	1.0147
East	Zhejiang	1.1197	0.9762	1.1472	1.0000
East	Fujian	1.1495	1.0309	1.1293	1.0000
East	Shandong	1.1607	1.0666	1.1373	0.9938
East	Guangdong	1.1344	1.0000	1.1368	0.9979
East	Hainan	1.1301	1.0043	1.1255	1.0000
Central	Shanxi	1.1558	1.0433	1.1215	0.9783
Central	Jilin	1.1584	1.0488	1.1302	0.9799
Central	Heilongjiang	1.0605	0.9511	1.1165	0.9872
Central	Anhui	1.1747	1.0781	1.1209	0.9755
Central	Jiangxi	1.1893	1.0594	1.1211	1.0044
Central	Henan	1.1220	1.0007	1.1512	0.9758
Central	Hubei	1.1435	1.1014	1.1607	0.9735
Central	Hunan	1.1632	1.0397	1.1127	0.9938
West	Inner Mongolia	1.1286	1.0621	1.1897	0.9578
West	Guangxi	1.1588	1.0399	1.1780	1.0048
West	Chongqing	1.2257	1.0364	1.1644	1.0274
West	Sichuan	1.1508	0.9902	1.1427	1.0261
West	Guizhou	1.1653	1.0085	1.1452	1.0306
West	Yunnan	1.0674	0.9509	1.1414	1.0114
West	Shaanxi	1.0809	0.9694	1.1248	1.0175
West	Gansu	1.0822	1.0207	1.1209	0.9800
West	Qinghai	1.0225	0.9270	1.1353	0.9817
West	Ningxia	1.1969	1.0141	1.1507	1.0274
West	Xinjiang	1.0454	0.9392	1.1436	0.9943
	Eastern China	1.1524	1.0241	1.1368	0.9988
	Central China	1.1459	1.0403	1.1294	0.9836
	Western China	1.1204	0.9962	1.1488	1.0054
	Whole China	1.1389	1.0181	1.1392	0.9972

**Table 4:** Global *Moran’s I* of provincial TCPI.

Year	2000–2001	2001–2002	2002–2003	2003–2004	2004–2005
*Moran’s I*	0.114 *	0.163 **	0.165 **	0.191 ***	0.225 ***
	[1.723]	[2.293]	[2.303]	[2.647]	[3.061]
Year	2005–2006	2006–2007	2007–2008	2008–2009	2009–2010
*Moran’s I*	0.269 ***	0.271 ***	0.287 ***	0.344 ***	0.265 ***
	[3.571]	[3.591]	[3.793]	[4.491]	[3.557]
Year	2010–2011	2011–2012	2012–2013	2013–2014	2014–2015
*Moran’s I*	0.256 ***	0.236 ***	0.220 ***	0.212 ***	0.211 ***
	[3.401]	[3.186]	[3.002]	[2.871]	[2.864]

Figures in parentheses are *Z* values. *, **, *** denote statistical significance levels at 10%, 5% and 1%, respectively.

**Table 5:** Estimation results at the national level using the four methods.

Type	Ordinary Static Panel Model (1)	Ordinary Dynamic Panel Model (2)	Static Spatial Panel Model (3)	Dynamic Spatial Panel Model (4)
τ (dynamic factor)		0.216 ***		0.102 ***
		[3.84]		[4.37]
ρ (spatial factor)			0.621 ***	0.003 ***
			[9.27]	[3.34]
ln*IS*	−0.035 ***	−0.029 ***	−0.047 ***	−0.043 ***
	[−3.53]	[−3.14]	[−3.43]	[−3.78]
ln*LH*	−0.128	−0.113 *	−0.092	−0.106 ***
	[−1.10]	[−1.70]	[−1.17]	[−2.93]
ln*SC*	0.061	0.067	0.055 *	0.053 **
	[0.82]	[1.28]	[1.77]	[2.05]
ln*OW*	−0.021	−0.011	−0.032	−0.025
	[−0.49]	[−0.87]	[−0.94]	[−1.06]
ln*EN*	−0.056 **	−0.054 ***	−0.057 ***	−0.048 ***
	[−2.32]	[−3.43]	[−3.72]	[−3.60]
ln*ECS*	−0.085 *	−0.092 *	−0.104 ***	−0.085 ***
	[−1.79]	[−1.74]	[−2.79]	[−3.52]
ln*FDI*	0.023	0.040	0.062	0.036
	[1.14]	[1.27]	[1.31]	[1.05]
ln*Tech*	0.040 ***	0.045 ***	0.024 ***	0.027 ***
	[5.87]	[5.24]	[5.38]	[5.61]
ln*Env*	0.024	0.026 *	0.036 **	0.035 **
	[1.21]	[1.83]	[1.99]	[2.21]
Cons	−0.502 ***	−1.224 ***	−0.342 ***	−0.154 ***
	[−2.98]	[−3.46]	[−4.65]	[−4.18]
Obs	450	420	450	420
LogL	123.736		150.285	174.363
LM-Lag test			(0.023)	(0.028)
Robust LM-Lag test			(0.054)	(0.070)
LM-Error test			(0.130)	(0.128)
Robust LM-Error test			(0.159)	(0.157)
Hausman test		(0.001)	(0.000)	(0.000)
System GMM test AR(1) test		(0.000)		(0.000)
AR(2) test		(0.252)		(0.233)
Hansen over-identification test		(1.000)		(1.000)

Figures in parentheses are *t* values. *, **, *** denote statistical significance levels at 10%, 5%, and 1%, respectively.

**Table 6:** Estimation results at the regional level.

Region	The Eastern China	The Central China	The Western China
τ (dynamic factor)	0.128 ***	0.107 ***	0.095 ***
	[5.13]	[4.52]	[3.87]
ρ (spatial factor)	0.007 ***	0.005 ***	0.002 ***
	[3.78]	[3.36]	[3.13]
ln*IS*	−0.043 **	−0.057 ***	−0.049 ***
	[−2.13]	[−3.95]	[−3.18]
ln*LH*	−0.119 ***	−0.123 ***	−0.104 ***
	[−3.07]	[−3.29]	[−2.76]
ln*SC*	0.048 ***	0.064 **	0.060
	[2.44]	[2.15]	[1.37]
ln*OW*	0.025	0.016 *	0.032
	[0.71]	[1.77]	[0.81]
ln*EN*	0.013 *	−0.047 ***	−0.079 ***
	[1.75]	[−3.68]	[−3.97]
ln*ECS*	−0.084 ***	−0.061 ***	−0.074 ***
	[−3.07]	[−3.92]	[−3.59]
ln*FDI*	0.025	0.045	−0.011 *
	[0.88]	[1.16]	[−1.77]
ln*Tech*	0.042 ***	0.030 ***	0.012 ***
	[6.37]	[5.68]	[4.35]
ln*Env*	0.049 ***	0.033 **	0.025 **
	[3.42]	[2.35]	[2.04]
Cons	−0.141 ***	−0.169 ***	−0.157 ***
	[−3.43]	[−4.64]	[−4.50]
Obs	154	112	154
LogL	73.335	51.274	69.208
LM-Lag test	(0.023)	(0.027)	(0.019)
Robust LM-Lag test	(0.048)	(0.066)	(0.040)
LM-Error test	(0.130)	(0.149)	(0.098)
Robust LM-Error test	(0.152)	(0.181)	(0.116)
Hausman test	(0.001)	(0.000)	(0.001)
System GMM test AR(1) test	(0.013)	(0.020)	(0.018)
AR(2) test	(0.248)	(0.281)	(0.273)

Figures in parentheses are *t* values. *, **, *** denote statistical significance levels at 10%, 5% and 1%, respectively.

**Table 7:** Estimation results of robustness test.

Type	The Whole China	The Eastern China	The Central China	The Western China
τ (dynamic factor)	0.104 ***	0.125 ***	0.108 ***	0.095 ***
	[4.53]	[5.17]	[4.28]	[3.62]
ρ (spatial factor)	0.005 ***	0.007 ***	0.004 ***	0.001 ***
	[3.63]	[4.08]	[3.26]	[2.86]
ln*IS*	−0.045 ***	−0.043 **	−0.056 ***	−0.047 ***
	[−3.86]	[−2.15]	[−3.87]	[−3.24]
ln*LH*	−0.104 ***	−0.119 ***	−0.120 ***	−0.097 ***
	[−2.92]	[−3.16]	[−3.38]	[−2.73]
ln*SC*	0.051 **	0.048 ***	0.059 *	0.052
	[2.03]	[2.45]	[1.76]	[1.24]
ln*OW*	−0.023	0.026	0.015 *	0.030
	[−1.22]	[0.64]	[1.74]	[0.69]
ln*EN*	−0.051 ***	0.014 *	−0.048 ***	−0.072 ***
	[−3.74]	[1.73]	[−3.67]	[−4.05]
ln*ECS*	−0.085 ***	−0.098 ***	−0.083 ***	−0.062 ***
	[−3.52]	[−3.27]	[−4.06]	[−3.71]
ln*FDI*	0.035	0.024	0.050	−0.009 *
	[1.17]	[0.73]	[1.08]	[−1.74]
ln*Tech*	0.025 ***	0.043 ***	0.028 ***	0.011 ***
	[5.52]	[6.31]	[5.60]	[4.18]
ln*Env*	0.034 **	0.048 ***	0.027 **	0.020 *
	[2.19]	[3.22]	[2.21]	[1.78]
Cons	−0.179 ***	−0.130 ***	−0.169 ***	−0.141 ***
	[−4.24]	[−3.47]	[−4.82]	[−4.37]
Obs	420	154	112	154
LogL	174.236	71.073	51.685	67.963
LM-Lag test	(0.026)	(0.026)	(0.027)	(0.020)
Robust LM-Lag test	(0.068)	(0.053)	(0.065)	(0.042)
LM-Error test	(0.124)	(0.135)	(0.147)	(0.099)
Robust LM-Error test	(0.150)	(0.157)	(0.176)	(0.118)
Hausman test	0.000)	(0.001)	(0.000)	(0.001)
System GMM test AR(1) test	0.000)	(0.016)	(0.021)	(0.021)
AR(2) test	(0.228)	(0.253)	(0.285)	(0.283)
Hansen over-identification test	(1.000)	(1.000)	(1.000)	(1.000)

Figures in parentheses are *t* values. *, **, *** denote statistical significance levels at 10%, 5% and 1%, respectively.
